# Methodology for the development of a taxonomy and toolkit to evaluate health-related habits and lifestyle (eVITAL)

**DOI:** 10.1186/1756-0500-3-83

**Published:** 2010-03-24

**Authors:** Federico Alonso, Carolyn O Walsh, Luis Salvador-Carulla, the eVITAL group

**Affiliations:** 1 Spanish Association for Research of Healthy Aging (Asociación Española para el Estudio Científico del Envejecimiento Saludable, AECES), Calle Infante Don Fernando 17, Antequera (Malaga) 29200, Spain; 2 Harvard Medical School, c/o Peabody Society, 260 Longwood Avenue, Boston, MA 02115, USA

## Abstract

**Background:**

Chronic diseases cause an ever-increasing percentage of morbidity and mortality, but many have modifiable risk factors. Many behaviors that predispose or protect an individual to chronic disease are interrelated, and therefore are best approached using an integrated model of health and the longevity paradigm, using years lived without disability as the endpoint.

**Findings:**

This study used a 4-phase mixed qualitative design to create a taxonomy and related online toolkit for the evaluation of health-related habits. Core members of a working group conducted a literature review and created a framing document that defined relevant constructs. This document was revised, first by a working group and then by a series of multidisciplinary expert groups. The working group and expert panels also designed a systematic evaluation of health behaviors and risks, which was computerized and evaluated for feasibility. A demonstration study of the toolkit was performed in 11 healthy volunteers.

**Discussion:**

In this protocol, we used forms of the community intelligence approach, including frame analysis, feasibility, and demonstration, to develop a clinical taxonomy and an online toolkit with standardized procedures for screening and evaluation of multiple domains of health, with a focus on longevity and the goal of integrating the toolkit into routine clinical practice.

**Trial Registration:**

IMSERSO registry 200700012672

## Background

As life expectancy continues rising [1], chronic diseases are becoming increasingly prominent causes of morbidity and mortality [2,3]. In high-income countries, most leading causes of both death and disability are noncommunicable disorders [2], many of which have well-established, modifiable risk factors [4-6]. After adjusting for age, these disorders cause an even greater loss of disability-adjusted life years in low- and middle-income countries [2]. Enhanced ability to identify at-risk individuals and modify related behavior would decrease morbidity and mortality worldwide.

The knowledge base regarding behaviors that promote healthy, disability-free aging continues to grow. Recent examples include prospective cohort studies about cognitive effects of alcohol intake [7] and adherence to a Mediterranean diet [8,9]. Unfortunately, evidence of health benefits of specific habits does not always translate to adoption of these behaviors. For example, in a prospective cohort study of more than 80,000 women, only 3% fit all five criteria--not smoking, body mass index <25, consuming ≥ 0.5 alcoholic drinks per day, ≥ 30 minutes of physical activity per day, and dietary score within the top 40% of the cohort--that placed them at lowest risk for cardiovascular disease; if all the women had met these criteria, cardiovascular events would have decreased by an estimated 82% [10].

Longevity medicine has been proposed as a proactive approach to extending healthy life expectancy and preventing chronic disease, beginning in the midlife period of 40-65 years of age [11]. It promotes an integrated model of health-related habits (HrH), with equal consideration of behaviors that improve and that compromise health. For most patients, primary care is the most appropriate setting within the health care system to begin addressing HrH. While ongoing research is investigating innovations to incorporate health promotion into primary care [12,13], providers report barriers such as minimal time with each patient, lack of policy-level prioritization of health promotion, and the need to address numerous risk factors [14]. With these limitations in mind, we set out to create an online toolkit with a formalized system to evaluate HrH, for incorporation into varying levels of health care. Toolkit development was framed by a new taxonomy of HrH, analogous to those used to classify diseases and other health determinants (e.g. taxonomy of medical errors [15]). Our focus was on the Spanish population. In this report, we describe the methodology used to develop the HrH taxonomy and accompanying toolkit, eVITAL.

## Methods

### Setting

This study was conducted in Antequera, Spain, by the Spanish Association for the Scientific Study of Healthy Aging (AECES), in a project funded by the Aging Institute (IMSERSO) of the Spanish Ministry of Health and Social Policies http://www.imsersomayores.csic.es/.

### Description of the Method

**Figure 1:**
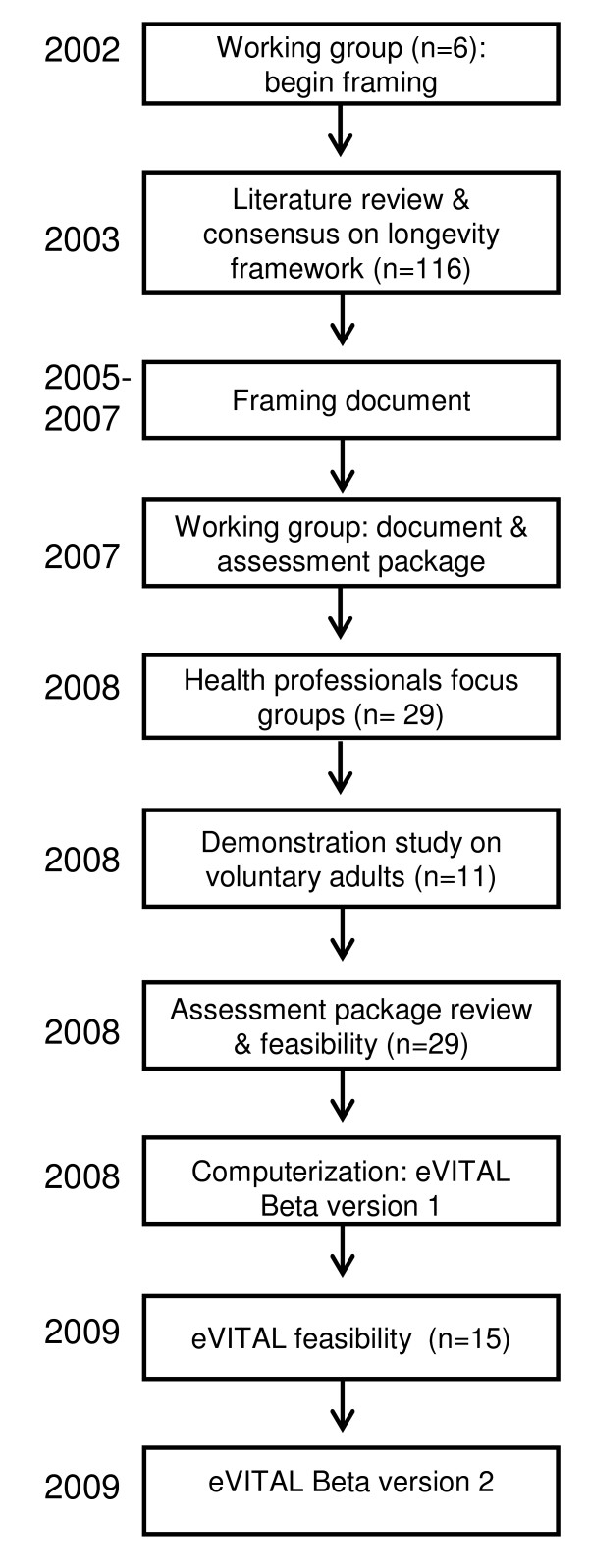
**Timeline of the eVITAL project**.

This study organizes information about HrH using longevity as the endpoint, with a four-phase mixed qualitative approach combined with frame analysis, feasibility and demonstration techniques to develop a toolkit usable in primary care. The study occurred over 8 years, as shown in Figure 1.

#### Phase 1: Assessment package development

Phase 1 consisted of a 4-pass approach to develop a taxonomy of HrH and compile a series of questionnaires and tests of HrH concepts that can be used clinically. A variety of expert groupings was employed during this phase (Table 1).

**Table 1:** Working Group Members

Name	Degree	Education/Training	Expert Field	Gender
Luis Salvador-Carulla (LSC)	MD, PhD	Psychiatry	Mental Health	Male

José Ricardo Cabo (JRC)	MD, PhD	Internal Medicine/Endocrinology	Diet	Male

Rafael Gómez (RG)	MD	General Medicine	Sleep and sexuality	Male

Carlos de Teresa (CT)	MD, PhD	Sports Medicine	Exercise	Male

Antonio Cano (AC)	MD, PhD	Obstetrics & gynecology	Gender	Male

Federico Alonso (FA)	MD, PhD	General Medicine	Disability, Health and social policy	Male

*Pass 1*-The goal of Pass 1 was to use frame analysis to create a document framing issues of HrH as they relate to the desired toolkit. Frame analysis is a broadly defined method of enumerating and defining ideas and themes within a larger topic that is particularly useful for defining new concepts [16]. It was applied here because, in spite of a long history of research and focus on healthy habits, there has been no previous attempt to operationalize and classify these health constructs and to build a related clinical toolkit.

The working group defined a set of key concepts in aging with their related domains, and identified relevant scientific references. Two core members (LSC, FA) coordinated the literature review. They contacted 116 experts in longevity, aging, and related fields. These experts produced a critical review of each concept using a standard procedure including Medline search. The working group reviewed the results and organized the international 2003 Andalusia Longevity Forum to discuss them with main experts in the field [17]. The resulting knowledge base was published [11]. The final key HrH domains identified were physical activity, diet, cognition/mental activity, sleep, psychosocial vitality, including sexuality, and risk behaviors, including drug and alcohol abuse. Reviews of these HrH were updated to include existing technical documents of IMSERSO and used to create the framing document.

*Pass 2*-In Pass 2, the framing document underwent a critical revision by the working group using a nominal group technique [17]. The panel defined subdomains and dimensions of the key domains identified in Pass 1, and selected questionnaires and other tools for history-taking and examination to be included in the formalized assessment package. Preference was given to instruments previously validated in Spanish. The group was moderated by one of the authors (FA), whose role was to ensure that rigorous criteria were used to select evaluation tools and to take notes on the proceedings, which were later presented to participants for comments and corrections.

*Pass 3- *In Pass 3, the framing document and evaluation system were further developed through a series of multidisciplinary focus group meetings. There were 4 focus groups, each moderated by members of the working group with additional expert members (Table 2). An invitation to participate was sent to 80 participants of the Andalusia Longevity Forum. Focus group members included the 23 who accepted the invitation. These groups held a series of in-person discussions, teleconferences, and personal communications. Each group was charged with adding to the existing literature review and the proposed definitions that pertained to its particular area of focus, to review the proposed clinical evaluation tools, and to prioritize areas within its domain.

**Table 2:** Composition of Expert Focus Groups

Topic	Moderators	Rapporteur	Group Member Backgrounds
Cognition	LSC	FA	Clinical psychology (2), geriatrics, neurology, neuropsychology

Vitality and stress	LSC	FA	Clinical psychology (2), gynecology, psychology of sexuality, psychiatry, sexuality, urology

Sleep	RG	FA	Clinical psychology, neurophysiology, sleep medicine

Diet, exercise, & risk behaviors	JRC	FA	Clinical psychology (2), nursing, physical activity, otorhinolaryngology, primary care (2), public health

Composition of multidisciplinary focus groups by area of expertise. For fields represented more than once within a group, the number of participants is indicated in parentheses. With the exception of the moderator of the first two groups and the rapporteur, no person is represented more than once. In total, 29 individuals participated.

*Pass 4*-In this final Pass, the framing document and evaluation package were returned to the working group, revised based on synthesis of focus group feedback, and resent to the focus groups for a final round of comments.

#### Phase 2: Demonstration Study

The second phase was a qualitative demonstration study involving 11 adult volunteers, recruited from the community of Antequera through a health spa mailing list and website posting. Males and females aged 40-64 were included. Exclusion criteria included disabling chronic illness and being a current patient of a member of the working group or focus groups. Volunteers were aware of the goal of creating an online toolkit and of the professional backgrounds of the investigators. Evaluation occurred in private at a local health spa. Parts of the evaluation requiring little or no training were self-administered, while more involved items were administered by members of the working group and expert panel previously trained with that tool. After completing the assessment package, study participants were asked to anonymously answer 5 open-ended questions (Additional File 1).

Results of the demonstration study were provided to members of the working group and focus groups and used to revise the assessment package. The final version of eVITAL was organized into 4 levels of increasing resource use: self-assessment (Level 0), basic primary care (Level 1), intensive primary care (Level 2), and specialty care (Level 3).

#### Phase 3: Computerization of eVITAL

The assessment package was transformed into an electronic toolkit by Conexanet SL, an information technology business.

#### Phase 4: Feasibility Study

A feasibility study involving members of the working group and focus group was conducted based on the 3 criteria of applicability, acceptability or "user-friendliness", and practicality [18], assessed initially following the demonstration study and again after computerization. The questionnaire (Additional File 2) was based on previous feasibility studies by our research group [19]; the combination of numerical and open-ended questions was designed to maximize return rate while allowing respondents to make any relevant comments. Responses were not anonymous.

### Ethics

One member of the working group and one member of an expert panel, both independent of the funding source, oversaw the ethical conduct of the study. The study complied with the Helsinki Declaration. Ethical approval for the study was granted by the Asociación PSICOST, an organization dedicated to the provision of services for people with disabilities in Spain. Demonstration study participants provided written informed consent.

### Data management and analysis

#### eVITAL demonstration study

Data from the volunteers were coded and maintained in a de-identified database. Procedures for maintaining privacy and security were registered with the General Data Protection Registry of the Spanish Agency of Data Protection, in accordance with Spanish Law of Data Protection 15/1999 (LOPD) [20]. There were 5 individuals charged with managing the results of the demonstration study: 1 for risk habits, vitality & stress, cognition, and mental health, 1 for exercise, 1 for diet, 1 for all other aspects, and 1 who oversaw all categories.

#### eVITAL feasibility study

Expert responses to the feasibility questionnaire were compiled and summary statistics were performed. Questions with particularly low scores were considered areas of concern warranting further review. Responses to the open-ended questions were evaluated for trends. Experts who suggested changes to the toolkit were contacted by one of the authors for further discussion.

### Toolkit validity

Whenever possible, evaluation items were selected that had been previously validated in Spanish. Items without previous Spanish validation were included with the intention of creating data registries for future analysis.

## Discussion

Longevity medicine is unique in its focus on health promotion and prevention and its consideration of both individual and public health perspectives [11]. In spite of available evidence and awareness of the contribution of HrH to longevity, and mounting literature on intervention approaches to HrH [14], there has been no attempt to provide a clinical taxonomy of HrH until now. Unlike many intervention studies and existing toolkits which focus on single health issues [21,22], this project considers the complex relationships between HrH in individual lifestyles. Our target demographic was middle-adulthood, to allow for identification of risk factors while the benefits of behavior change have years to accumulate [23]. Our protocol formalizes evaluation procedures, progressing from a self-administered screen to specialty care. In clinical practice, results from Levels 0 and 1 would alert clinicians to at-risk patients who merit in-depth evaluation. Our goal was not to replace clinical judgment, but rather to inform and guide evaluation of HrH.

There are several remaining steps before eVITAL is ready for integration into routine clinical care. The demonstration study was conducted on the non-computerized assessment package, and the feasibility analysis on the Beta-1 version of the toolkit. An equivalent assessment must be completed with the computerized Beta-2 version, and the results used for further revision. Also, while the protocol included the demonstration study of healthy volunteers in a non-medical setting, a similar assessment has yet to be completed in medical practice.

Our ultimate aim is to incorporate free use of eVITAL into the primary care system in Spain and to allow improvements and research by clinicians and other health professionals, as well as to establish "semantic interoperability" with other health websites. Further work is needed to delineate appropriate treatment algorithms based on evaluation results and to determine cost-effectiveness of various sections of the toolkit. Language-related needs include validation studies of the inventories not previously available in Spanish and assessments of whether the toolkit is culturally appropriate for Spanish-speaking populations outside of Spain. The toolkit is not meant to be static, but can be updated and adapted for use in specific clinical populations.

The recent dramatic increase in available health information has posed an enormous challenge to health knowledge management, especially in areas like HrH that lack a consensus on taxonomical framework, and where international cooperation is needed to build a comprehensive knowledge base. In this protocol, we used forms of community intelligence to begin addressing these issues for HrH. As we move forward, we hope to develop an information model to formally represent components of the project, to incorporate input from broader forms of community intelligence, similar to GeneWiki in genetics [24], and to increase usability of the toolkit in clinical practice.

## Competing interests

The authors declare that they have no competing interests.

## Authors' contributions

LSC designed the study, carried out the literature review, and participated in the working group. FA coordinated data collection, carried out the literature review, and participated in the working group. CW wrote the manuscript. All authors read and approved the final manuscript.

## Supplementary Material

Additional file 1 **Demonstration Study Questionnaire**. Written questionnaire given to adult volunteers during the Phase 2 demonstration study of the eVITAL toolkit evaluating health-related habits. Click here for file

Additional file 2 **Feasibility Study Questionnaire**. Questionnaire sent to experts during the Phase 4 feasibility study of the eVITAL toolkit evaluating health-related habits. Click here for file
