# Supplementary material for: Methodology for the development of a taxonomy and toolkit to evaluate health-related habits and lifestyle (eVITAL)
Source: BMC Res Notes. 2010 Mar 24;3:83. doi: 10.1186/1756-0500-3-83 (PMC3003271; doi:10.1186/1756-0500-3-83)
Supplement: Additional file 1 — Demonstration Study Questionnaire. Written questionnaire given to adult volunteers during the Phase 2 demonstration study of the eVITAL toolkit evaluating health-related habits. [file 1756-0500-3-83-S1.DOC]

## Additional File 1 - Demonstration Study Questionnaire

| 1. What is your general impression of the study that you have completed?2. What was your impression of the study personnel?3. What would you change to improve your impression of the study?4. Did completing the study make you tired or fatigued?5. Would you pay for this evaluation? |
| --- |

**Additional File 1. Written questionnaire given to adult volunteers during the Phase 2 demonstration study of the eVITAL toolkit evaluating health-related habits.**
