# Supplementary material for: Methodology for the development of a taxonomy and toolkit to evaluate health-related habits and lifestyle (eVITAL)
Source: BMC Res Notes. 2010 Mar 24;3:83. doi: 10.1186/1756-0500-3-83 (PMC3003271; doi:10.1186/1756-0500-3-83)
Supplement: Additional file 2 — Feasibility Study Questionnaire. Questionnaire sent to experts during the Phase 4 feasibility study of the eVITAL toolkit evaluating health-related habits. [file 1756-0500-3-83-S2.DOC]

## Additional File 2 – Feasibility Study Questionnaire

| *Part A*: Evaluator Characteristics |
| --- |
| A1. Name  A2. Age  A3. Gender  A4. Education   1. Advanced degree (e.g. physician, psychologist, nurse) 2. Undergraduate degree (e.g. social worker) 3. No degree/other   A5. Profession/job (describe)  A6. Years of professional experience |
| *Part B*: General Feasibility of eVITAL questionnaire  Please complete the following questions regarding the utility of the eVITAL questionnaire as a whole. (Questions B1-B11 are on a 4-point scale, from very high to very low) |
| B1. Applicability- Do you think the content of the eVITAL questionnaire is meaningful in the measurement of the health status of people over 55 years old?  B2. Accuracy & simple reliability- Do you think the people being interviewed would be able to provide reliable and exact information?  B3. Acceptability (social/psychological)- Do you think the questionnaire is acceptable for this type of interview?  B4. Ease of use- In your opinion, in general how difficult is the questionnaire?  B5. In your opinion and experience, the level of training needed to complete the questionnaire is:  B6. In your opinion, the time necessary to complete it is:  B7. In your opinion, the complexity of coding the data is:  B8. In your opinion, the complexity of interpreting the data is:  B9. Practicality- Is it possible to use this questionnaire in routine practice, for monitoring and tracking?  B10. Is it possible to use this questionnaire for general health studies/databases?  B11. Cost/benefit effectiveness- What is the general benefit of the eVITAL questionnaire in relation to the difficulty or cost of collecting the information?  B12. Suggestions for changes or corrections to the toolkit  B13. Would you organize the sections differently?  B14. Other comments |
| *Part C*: Feasibility of the sections of the eVITAL questionnaire  Please complete the following questions for each section of the questionnaire (cognition, vitality & stress, sleep, health-related behaviors: diet, exercise, risk behaviors, substance use). Keep in mind which indicator or health domain you are evaluating in each section. Focus on the sections that, in your experience, have issues with utility/feasibility. (Questions B2-B12 are on a 4-point scale, from very high to very low). |
| C1. Name of the section:  C2. Applicability- Is the content of the section meaningful for the indicator that it is supposed to measure?  C3. Accuracy & simple reliability- Do you think the people being interviewed would be able to provide reliable and exact information for this section?  C4. Acceptability (social/psychological)- Do you think this section is acceptable to the people being interviewed?  C5. Ease of use- In your opinion, what is the general difficulty of this section?  C6. In your opinion, the level of training needed to complete this section is:  C7. In your opinion, the time necessary to complete this section is:  C8. In your opinion, the complexity of interpreting the data from this section is:  C9. Practicality- What is the general level of practicality of this section (the degree to which the items are clinically applicable)?  C10. Practicality- Is it possible to use this section in routine practice, for monitoring and tracking?  C11. Is it possible to use this section for general health studies/databases?  C12. Cost/benefit effectiveness- What is the general benefit of this section in relation to the difficulty or cost of collecting the information?  C13. Is there any important item or domain that has not been included?  C14. Is there any irrelevant or unimportant item or domain that should be eliminated?  C15. Would you organize the items/sections differently? |

**Additional File 2. Questionnaire sent to experts during the Phase 4 feasibility study of the eVITAL toolkit evaluating health-related habits.**
